# Aesthetic perception of maxillary incisor inclination in smiling profile of different facial divergences

**DOI:** 10.1038/s41405-025-00387-9

**Published:** 2025-12-08

**Authors:** Mojgan Shavakhi, Amirmohammad Yavari, Kioumars Tavakoli Tafti

**Affiliations:** 1 Department of Orthodontics, Dental Research Center, Dental Research Institute, School of Dentistry, Isfahan University of Medical Sciences, Isfahan, Iran; 2 Graduate Student, Students Research Committee, School of Dentistry, Isfahan University of Medical Sciences, Isfahan, Iran

**Keywords:** Aesthetic dentistry, Orthodontics

## Abstract

**Objectives:**

This study aimed to evaluate the effect of maxillary incisor inclination on the aesthetic perception of the smiling profile in different facial divergences.

**Materials and methods:**

A smiling profile photograph of a young woman was digitally altered to obtain three image series with different facial divergences. In each series, the inclination of the incisor teeth was changed in 5° intervals within a range of −15° to +15°. The 21 final images were submitted to 45 orthodontists and 45 laypeople to be rated based on the visual analogue scale.

**Statistical analysis:**

The data were analysed with the independent *t*-test, repeated-measures ANOVA, and Pearson’s correlation coefficient.

**Results:**

The most attractive images for orthodontists were −5° in posterior divergence, 0° in neutral divergence and +10° in anterior divergence. While for laypeople, they were −10° in posterior divergence, 0° in neutral divergence and +5° in anterior divergence. In the posterior and anterior divergent faces, laypeople preferred more negative inclinations compared to orthodontists, but in all three faces, both groups chose the same inclinations as the most unattractive.

**Conclusions:**

A harmony between the inclination of the maxillary incisor teeth and facial divergence leads to more aesthetic outcomes in such a way that in posterior divergence faces, negative inclinations, in anterior divergence faces, positive inclinations and in neutral divergence faces, incisors without inclination seemed to be more attractive. Also, laypeople tended to prefer more retroclined incisors than orthodontists.

## Introduction

Aesthetic improvement is one of the most important goals of orthodontic treatment [1, 2] and is one of the main reasons patients seek orthodontic treatment [3]. In this line, 80% of patients are motivated to refer themselves or their children for orthodontic treatment for this reason. [4, 5]. The smile is one of the most important facial expressions and an essential component of emotions such as friendship, satisfaction and happiness, which plays a critical role in facial aesthetics [6, 7]. Kerns et al. showed that the attractiveness score of identical smiles in the profile view is higher than in the frontal view [8]; therefore, it is essential to consider both views in clinical examinations and orthodontic treatment planning [9].

An important factor in the beauty of a smile in the profile view is the inclination of the maxillary incisor teeth [3, 7, 10]. During orthodontic treatment, incisor teeth are aligned using torque forces to achieve maximum aesthetics [11, 12]. In order to achieve maximum facial beauty in a person, irrespective of numerical measurements, one should pay attention to the harmony between the components [13]. In this context, the relationship between soft and hard tissue becomes especially important [2].

Although the concept of beauty is subjective, efforts have been made to achieve a formula for this matter for a long time [14]. Studies to determine the ideal inclination of maxillary incisors to achieve the most beauty started in 1987 when Philippe declared that the buccal surface of the maxillary incisor teeth should be vertical and parallel to the frontal plane of the face [15]. Since then, many studies have evaluated the effects of maxillary incisor inclination on smile aesthetics. These studies have reported different incisor inclinations, such as lingual [2, 16, 17], vertical [10, 18], or buccal [1, 19], as the ideal incisor inclination that can result in the most beautiful smile. Furthermore, in some studies, different raters, such as orthodontists and laypeople, deemed different inclinations the most attractive [1, 15].

On the other hand, different facial divergences can be seen in the profile view, which is defined by the inclination of the lower face relative to the forehead and include anterior, neutral, or posterior divergence [20]. Although convex or concave facial profiles are considered problematic, a straight profile is considered normal regardless of the divergence. Therefore, treatment for facial divergence is not suggested, and during orthodontic treatment, other facial features and elements, including incisor inclination, are adjusted according to the facial type [21].

Although studies have investigated the effect of the inclination of maxillary incisor teeth on the aesthetics of the facial profile, to the best of our knowledge, no study has considered facial divergence as an independent factor. Therefore, this study aimed to evaluate the effect of maxillary incisor inclination on the aesthetic perception of the smiling profile in different facial divergences.

## Materials and methods

This cross-sectional study was approved by the University’s Research Ethics Committee (IR.MUI.RESEARCH.REC.1400.376) and was conducted at the Isfahan University of Medical Sciences from January to June 2022.

Documents of the patients whose orthodontic treatment was finished in the Orthodontics Department were reviewed, and a 16-year-old female subject was selected and recalled for this study. The study was thoroughly explained to the patient, and written consent was obtained from both the patient and her parents. It was mentioned in the consent form that the photos will be published in a recognisable form. The subject was selected based on the following criteria, confirmed by two orthodontists: Neutral divergence of facial profile and even vertical third of the face (defined by Legan and Burstone [22]); normal soft tissue anatomy, including lips, chin and nose, and no history of aesthetic surgery, including rhinoplasty; class 1 dental occlusion (molar and canine) and skeletal relationship (ANB angle = 0–4°) [23]; the presence of the maxillary anterior teeth from central incisor to at least canine in the profile view of the smile with an appropriate gingival show (2–4 mm); soundness of the incisor teeth with normal anatomy; no history of dental trauma, missing or restoration of anterior teeth; and proper inclination (U1 to FH = 106–116°) and anteroposterior position of the maxillary incisor teeth in relation to the forehead as defined by Andrews [24].

**Fig. 1:**
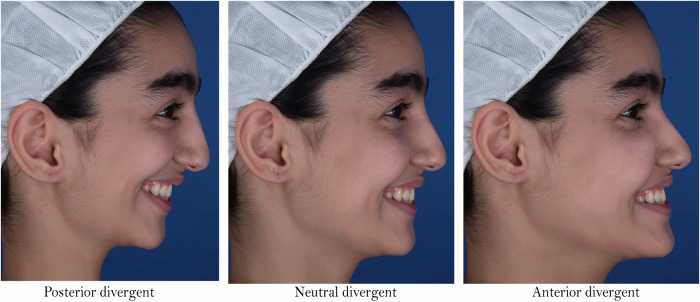
Digitally altered facial divergence of the original image. From left to right: posterior divergence, neutral divergence (initial photo) and anterior divergence.

The selected patient was recalled for taking the photographs. A blue screen was used as the background, with a vertical line indicating the actual vertical direction. To obtain photographs in natural head position, the subject was asked to stand in front of a mirror and look into her eyes. Several photographs were taken from the profile view at rest and smile using a NIKON D750 camera (Nikon Corporation, Tokyo, Japan) on a tripod at a distance of 5 feet from the subject. The lighting was provided by a mixture of natural light and white lamplight. Before calling the patient, she was not informed about taking pictures while smiling to prevent the patient from practicing in front of a mirror. The best smiling photograph was selected and edited using Photoshop software (Adobe Photoshop CS, version 8.0; Adobe Systems, San Jose, Calif) to obtain the desired changes. To create the anterior and posterior divergent types, considering N-pog’ as a reference, the anterior part of the face below the Nasion was rotated by 10 degrees with Nasion as the centre of rotation (10 degrees backward for posterior divergent and 10 degrees forward for anterior divergent) [21]. () Then the soft tissue of the face, which lost its uniformity with the changes, was retouched to create a natural appearance. This rotation was applied only to the anterior part of the patient’s face below the eyes so that the visual axis remained unchanged. The quality of the photographs was checked and approved by two orthodontists to ensure realistic views (Fig. 1).

**Fig. 2:**
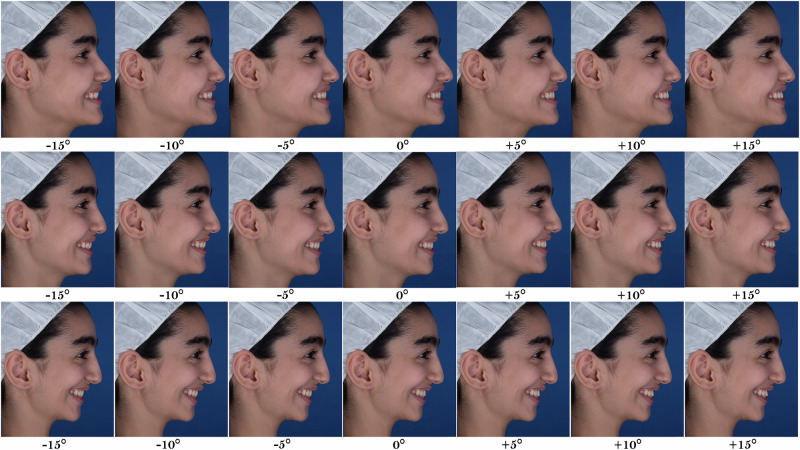
Digitally altered incisor teeth inclination in each facial divergence. Each row represents one group of images (from top to bottom: posterior divergence, neutral divergence and anterior divergence). In each row, the inclination increases from the left (−15°) to the right (+15°) in 5° intervals.

In the next step, the inclination of the maxillary incisor teeth was changed in each series of these images. In order to create the desired inclination of the maxillary incisors, central and lateral incisors were selected in the image and rotated from −15° to +15° in 5° intervals (positive rotation in the counterclockwise direction). This rotation angle was considered compared to the original photograph, and the rotation of the face that caused the rotation of the incisor teeth was also calculated in this angle change (e.g. the primary image of the posterior divergence was considered the −10° inclination image). Then, incisor teeth were moved in the anteroposterior direction so that the central incisors’ most prominent (buccal) points did not change relative to the primary position. If necessary, the teeth and the gum were retouched to make the photograph look natural. Finally, after making the desired changes, 21 photographs (three series of 7 photographs) were obtained (Fig. 2).

### Photograph evaluation

The raters consisted of two groups: 45 orthodontists and 45 laypeople. All participants consented to take part in the study. The sample size was calculated according to Ghaleb et al. [19], considering a standard deviation of 19.42, absolute precision of 11.43, *α* = 0.05 and *β* = 0.2. The orthodontists were selected with a simple random sampling method from the list of orthodontic specialists working in the three main cities of Iran with at least five years of clinical experience. Laypeople were also selected randomly from the patients referred to the Faculty of Dentistry of the same cities for non-aesthetic treatments, who were 18–50 years of age. Exclusion criteria were a history of orthodontic treatment, facial surgery, or any cosmetic treatment, facial deformity or trauma and education or employment in dentistry or art.

In order to score, the photographs were printed in a dimension of 10 × 15 cm and placed in an album in three groups of facial divergence. The order of placing the photographs in each group was determined randomly, and a code was assigned to each. The participants were asked first to view all the photographs once. Then, they were asked to view the photographs from the beginning and, this time, rate the smile’s attractiveness in each photo using the visual analog scale. It was not possible to go back during the rating. The raters were given a checklist containing 21 lines measuring 100 mm long, graded from 0 (least attractive) to 100 (most attractive), and the code of each photograph was written next to each line.

In order to measure the reliability of the ratings, 10% of the participants (five participants from each group) were randomly selected and asked to rate the images again after 2 weeks.

### Statistical analysis

In order to perform statistical analyses, the data were fed into SPSS 26 software and described using means and standard deviations. To assess the normality of data, Kolmogorov-Smirnov test was used which showed normal distribution. The aesthetic scores of the same images were compared between the two groups using the independent t-test. Repeated-measures ANOVA was performed to compare the differences between the aesthetic scores of the images in each rater group. Pearson’s correlation coefficient was used to determine the correlation between the aesthetic scores and both genders and the age of the raters. A significance level of 0.05 was considered. Intraclass correlation coefficient (ICC) was used, with a 95% confidence interval, to test the reliability of the scores.

## Results

The evaluators consisted of 45 orthodontists (25 women, 19 men) with a mean age of 37.11 years (SD = 9.17) and 45 laypeople (27 women, 17 men) with a mean age of 32.84 (SD = 10.03). It should be noted that the average professional experience in the orthodontists’ group was 10.75 (SD = 9.57) years. There was no significant difference in judging the incisor inclination’s effect on smile attractiveness between the men and women, and also, there was no significant relationship between the age and the scores.

The ICC was 81.8% (confidence interval: 95%; lower bound: 68.7%; upper bound: 91.2%), indicating a high level of agreement between judges and the reliability of the scores.

The average aesthetics scores given by each group of evaluators to the images of different facial divergence are presented in Table 1 (posterior divergent), Table 2 (neutral divergent), and Table 3 (anterior divergent).

**Table 1:** The average aesthetic scores to the posterior divergent images.

Incisor inclination	Laypeople	Orthodontists	*p*-value
	Mean (±SD)	Mean (±SD)	
−15°	64.09 (±24.94)	47.93 (±19.69)	0.001^*^
−10°	70.91 (±25.13)	57.61 (±21.19)	0.009^*^
−5°	66.75 (±22.92)	59.30 (±20.10)	0.109
0°	48.00 (±23.33)	53.07 (±19.59)	0.273
+5°	37.95 (±20.55)	48.84 (±16.57)	0.008^*^
+10°	26.98 (±17.69)	40.20 (±18.08)	0.001^*^
+15°	16.36 (±16.15)	33.52 (±19.86)	0.000^*^

^*^p-value < 0.05.

**Table 2:** The average aesthetic scores to the neutral divergent images.

Incisor inclination	Laypeople	Orthodontists	*p*-value
	Mean (±SD)	Mean (±SD)	
−15°	61.93 (±23.72)	49.07 (±20.26)	0.008^*^
−10°	66.36 (±20.69)	57.16 (±20.11)	0.037^*^
−5°	72.61 (±21.25)	66.98 (±18.07)	0.184
0°	75.11 (±20.50)	72.39 (±14.20)	0.470
+5°	67.09 (±19.96)	70.20 (±16.32)	0.425
+10°	48.80 (±21.47)	61.93 (±17.59)	0.020^*^
+15°	33.84 (±24.78)	42.39 (±20.95)	0.084

^*^p-value < 0.05.

**Table 3:** The average aesthetic scores to the anterior divergent images.

Incisor inclination	Laypeople	Orthodontists	*p*-value
	Mean (±SD)	Mean (±SD)	
−15°	29.82 (±20.42)	29.20 (±18.20)	0.882
−10°	36.59 (±21.74)	36.95 (±19.92)	0.935
−5°	42.66 (±26.70)	45.07 (±20.78)	0.638
0°	46.82 (±22.80)	53.14 (±20.65)	0.177
+5°	56.36 (±26.64)	52.93 (±21.53)	0.508
+10°	52.39 (±24.60)	54.11 (±22.20)	0.730
+15°	43.30 (±27.49)	47.50 (±21.23)	0.882

In the posterior divergent image series, orthodontists assigned the highest score to the smile with −5° inclination of incisors, while the laypeople assigned the highest score to −10°. Both groups of raters assigned the lowest scores to the incisor inclination of +15°. When comparing the aesthetic scores of the two groups of evaluators, significant differences were found in −15° (*p*-value = 0.001) and −10° (*p*-value = 0.009) of incisor inclination with higher scores assigned by laypeople and also in +5° (*p* = 0.008), +10° (*p* = 0.001) and +15° (*p* = 0.000) with higher scores assigned by orthodontists.

In the neutral divergent image series, both groups of evaluators rated the vertical position of incisors (0°) as the most attractive and the inclination of +15° as the least attractive. The aesthetic scores assigned by two rater groups were significantly different in the incisor inclination of −15° (*p* = 0.008) and −10° (*p* = 0.037), where laypeople assigned higher scores and in the inclination of +10° (*p* = 0.020), where the scores rated by orthodontists were higher.

In the anterior divergent image series, laypeople and orthodontists rated the incisors’ inclinations of +5° and +10° as the most attractive, respectively. According to both rater groups, the inclination of −15° had the lowest attractiveness score. No significant difference was found between the two groups of evaluators in any image of this series of images.

In each rater group, aesthetic scores of the images were compared one by one using repeated-measures ANOVA. Table 4 presents the images that had the same attractiveness score as the most attractive or the least attractive image.

**Table 4:** The most and the least attractive inclination of incisors and the inclinations with similar attractiveness in each series of images according to two rater groups.

Facial divergence	Raters group	The most attractive	As aesthetic as the most attractive	The least attractive	As aesthetic as the least attractive
Posterior divergent	Orthodontists	−5	−10 0	+15	**−**
	Laypeople	−10	−15 −5	+15	−
Neutral divergent	Orthodontists	0	+5	+15	−
	Laypeople	0	−5 +5	+15	−
Anterior divergent	Orthodontists	+10	0 +5	−15	−
	Laypeople	+5	+10	−15	−10

## Discussion

This study focused on profile-view smile aesthetics. Maxillary incisor inclination is a key factor influencing profile smile attractiveness, which should be considered in harmony with other facial components. In previous studies, the inclination of incisor teeth has been well investigated in straight profiles with neutral divergence; however, the results of these studies have been different and contradictory. In this study, in addition to neutral divergence, two other facial types, i.e. posterior and anterior divergence, were also considered and the inclination of incisor teeth was investigated in these three facial types. Both orthodontists and laypeople were included as evaluators to assess smile aesthetics. Laypeople were included because they represent the primary judges of aesthetics in real-life social interactions [25]. Although some studies employ only laypeople as raters, a reference for comparison is essential. Therefore, orthodontists were selected as the second group, given their clinical expertise in performing all treatment procedures.

The findings of this study showed that maxillary incisor inclination significantly affects individuals’ aesthetics in smiling profiles. This effect varied in different facial divergences. In faces with neutral divergence, the incisor teeth inclination of 0° was considered the most beautiful from the point of view of orthodontists, and the incisor inclination of +5° ranked second in terms of beauty. Similar to the findings of this study, most previous studies have shown that according to orthodontists, the vertical position of the incisor teeth (inclination of 0 degrees) is the most attractive [2, 10, 15, 18] in straight profiles with neutral divergence faces. However, in some other studies, the inclinations of +5° [1, 19], +15° [26] and −5° [27] have also been mentioned as the most beautiful inclinations of incisor teeth. Similar to orthodontists, laypeople in this study chose the 0° inclination of the incisor teeth as the most beautiful inclination and the −5° as the second most beautiful in the neutral divergence profile. Most previous studies have shown that laypeople consider −5° as the most beautiful incisor inclination [2, 15, 16, 26, 27]. However, in other studies, inclinations of 0° [1, 10, 18], +5° [15, 19] and −10° [17] have also been mentioned as the most beautiful inclination from the point of view of laypeople.

On the other hand, according to laypeople, the lowest attractiveness score in this study, as well as most previous studies [2, 15, 17–19], has been assigned to +15°. Similar to laypeople, orthodontists assigned the lowest score to the +15° and the −15° ranked the second least attractive. While different findings have been observed in previous studies, the incisor inclinations of −15° [18, 19, 27], −10° [1, 15] and +15° [2] have been considered as the most unattractive inclination of incisor teeth in straight profiles from the orthodontists’ viewpoint. Differences might be attributed to the different facial characteristics of the subjects or social and cultural differences among the raters.

In the posterior divergence face, retroclined incisors were chosen as the most attractive position of the incisor teeth. However, laypeople preferred slightly more retroclined incisors, so that the most attractive inclination was −10° according to laypeople and −5° according to orthodontists. On the other hand, the lowest scores were assigned to proclined incisors, and in the opinion of both groups, the incisal inclination of +15° was the most unattractive, similar to the results of the neutral divergent face. Also, similar to the results of the neutral divergent group, negative incisal inclinations were scored higher by laypeople; on the contrary, positive incisal inclinations were scored higher by orthodontists.

In the anterior divergence face, proclined and vertical incisors were preferred. Orthodontists chose +10° as the most attractive, while laypeople chose +5°. On the other hand, both groups selected −15° inclination as the least attractive. Unlike the neutral and posterior divergence faces, in the anterior divergence face, proclined incisors were preferred. Also, compared to laypeople, orthodontists preferred more proclined incisors, but there was no significant difference between the scores of these two groups in the anterior divergence images.

As mentioned earlier, in previous studies, facial divergence was not considered a factor that can affect the attractiveness of incisor inclination. However, in a study by Najafi et al. [1], the inclination of the incisors within a range of -10 to +10 was evaluated in relation to different anterior-posterior positions of the mandible in a male subject. Their results are somewhat consistent with the current study. Najafi et al.’s study showed that in faces with a prognathic mandible, an incisor inclination of −10° is the least attractive from the point of view of orthodontists and laypeople. Also, in the faces with retrognathic mandible, +10° inclination was the least attractive according to laypeople. These findings are partially consistent with the present study regarding anterior and posterior divergent profiles, respectively. However, other findings of these two studies are different due to the different nature of facial divergency and mandibular position.

Considering the findings of this study and previous studies, it seems that laypeople find slightly more negative incisal inclination more attractive than orthodontists. It is most obvious in the most attractive inclinations of posterior and anterior divergences, in which laypeople chose more negative inclinations (−10 and +5) compared to orthodontists (-5 and +10). However, both groups chose the same inclinations as the most unattractive in all three types of facial divergence. Also, between the two groups, some inclinations were rated significantly differently in posterior (−15°, −10°, +5°, +10° and +15°) and neutral (−15°, −10° and +10°) divergence faces. This difference of opinions between laypeople and orthodontists should be considered in orthodontic treatment, where the inclination of maxillary incisors may be altered, as it can cause clinical problems with the treatment outcomes and patients’ satisfaction. Also, the results of this study did not show any relationship between the age or gender of raters and their scores, similar to previous studies [1, 18, 19, 27].

This study showed that establishing harmony between incisor inclination and facial divergence leads to more aesthetics. In posterior divergence faces, the aesthetics score of negative inclinations was significantly higher. The aesthetics score of maxillary incisor teeth without inclination was highest in neutral facial divergence, and the positively inclined teeth in the anterior divergence face gained significantly higher aesthetics scores. Also, orthodontists and laypeople may have different preferences, which should be considered. It is important to prioritise patients’ opinions in treatment planning as far as it is logical and applicable. Also, the results of the present study can help orthodontists with clinical decisions. Considering the higher aesthetic score of negative inclinations in posterior divergent faces, extraction or lingual crown torque of maxillary anterior teeth, might be more applicable in such cases. While in anterior divergent faces, extractions should be evaluated more cautiously and keep the buccal inclination of the maxillary incisors. These results might differ in subjects or raters of other populations. Therefore, the current findings should be interpreted and generalised with caution. For further studies, it is suggested to evaluate the effect of maxillary incisors’ inclinations on smile aesthetics concerning other facial properties, genders and races.

## Conclusions

- The effect of incisor inclinations on the aesthetics of the smiling profile is related to individuals’ facial divergence, and clinicians should consider the facial divergence of the patients as a factor for adjusting the incisor inclinations to achieve more aesthetically pleasant outcomes.
- In posterior divergence faces, negative inclinations were considered more attractive than positive ones in anterior divergence faces. Moreover, the aesthetics score of maxillary incisor teeth without inclination (0°) was the highest in the neutral divergence faces.
- Laypeople prefer negative inclinations and find them more beautiful compared to orthodontists. This discordance by clinicians is critical because it can affect patients’ satisfaction with orthodontic treatments.
